# *Helicobacter pylori* CagA Disrupts Epithelial Patterning by Activating Myosin Light Chain

**DOI:** 10.1371/journal.pone.0017856

**Published:** 2011-03-21

**Authors:** Jonathan B. Muyskens, Karen Guillemin

**Affiliations:** Institute of Molecular Biology, University of Oregon, Eugene, Oregon, United States of America; Charité-University Medicine Berlin, Germany

## Abstract

*Helicobacter pylori* infection is a leading cause of ulcers and gastric cancer. We show that expression of the *H. pylori* virulence factor CagA in a model *Drosophila melanogaster* epithelium induces morphological disruptions including ectopic furrowing. We find that CagA alters the distribution and increases the levels of activated myosin regulatory light chain (MLC), a key regulator of epithelial integrity. Reducing MLC activity suppresses CagA-induced disruptions. A CagA mutant lacking EPIYA motifs (CagA^EPISA^) induces less epithelial disruption and is not targeted to apical foci like wild-type CagA. In a cell culture model in which CagA^EPISA^ and CagA have equivalent subcellular localization, CagA^EPISA^ is equally potent in activating MLC. Therefore, in our transgenic system, CagA is targeted by EPIYA motifs to a specific apical region of the epithelium where it efficiently activates MLC to disrupt epithelial integrity.

## Introduction

*Helicobacter pylori* is a Gram-negative bacterium that is estimated to infect over half the world's population [1], [2], [3], [4]. Virulent strains of the bacterium contain a genetic element called the CagA pathogenicity island (CagA PAI), which encodes components of a Type IV secretion system and the virulence factor, CagA [5]. CagA does not share homology with any known proteins, and therefore its mechanism of action has remained poorly understood. Much of what is known about CagA is through cell culture studies. Using cultured human gastric cells, it was shown that CagA is inserted into host cells through a type IV secretion system, and once inside the cell it is phosphorylated by Src kinases at tyrosines within repeated EPIYA motifs [5]. Phosphorylated CagA, in turn, ectopically activates the tyrosine phosphatase, SHP-2, a well-characterized protooncogene [3], [5].

CagA has been shown to alter the cytoskeleton of cultured cells. For example, in a cultured epithelial monolayer, individual CagA-expressing cells elongate, lose polarity, and migrate away from the monolayer, a process resembling an epithelial to mesenchymal transition [6]. SHP-2 activation by CagA causes cultured human gastric cells to elongate, a phenomenon referred to as “the hummingbird phenotype” [7]. Previously, we showed that CagA-induced cell elongation resulted from a failure of cell retraction and was not dependent on the RhoGTPases, Cdc42 and Rac1 [8]. For technical reasons we were unable to test whether another small RhoGTPase, RhoA, was involved in CagA-induced cell elongation in human gastric cells. However, the Drosophila homolog of RhoA, Rho1, is known to mediate retraction of the trailing edge of migrating hemocytes in the fly embryo [9] and RhoA, is active at the trailing edge of human neutrophils [10].

A key effector of RhoA in cell retraction is myosin light chain (MLC), a component of the hexameric motor protein, non-muscle myosin 2 (NMM2) [11], [12]. When RhoA is activated by its guanine nucleotide exchange factor, RhoGEF2, it activates Rho Kinase, which directly phosphorylates key serine and threonine residues on MLC [10]. Another kinase, Myosin Light Chain Kinase (MLCK) also phosphorylates MLC at the same serine and threonine residues [13]. Upon phosphorylation, MLC becomes active and uses actin as a substrate to induce cellular contraction [13]. For example, in the developing *D. melanogaster* eye epithelium, MLC-mediated apical actin constriction drives formation of a dynamic signaling center in the developing eye imaginal disc called the morphogenetic furrow (MF) [14]. A transgenic *D. melanogaster* expressing a constitutively active mutant form of MLC (MLC E20E21) has been generated in which the key phosphorylation sites, Ser20 and Thr21, were replaced with phosphomimetic glutamates [15]. Ectopic expression of this mutant in clones of eye imaginal disc cells causes the expressing cells to constrict apically and form an ectopic furrow [14]. In the mammalian intestine, transgenic expression of MLCK results in disrupted epithelial barrier function, causing broad immune activation and upregulation of cytokine expression [16].

To gain mechanistic understanding of CagA’s activity in complex tissues, our group developed a *D. melanogaster* CagA transgenic model [17]. Reagents for modulating the Rho/MLC pathway are readily available in *D. melanogaster,* making it an attractive model for assessing potential interactions with CagA. Because of the availability of a large collection of Gal4 lines, it possible to express our UAS:CagA transgene with exquisite spatial and temporal resolution at all stages of development. Furthermore, transgenic expression of CagA with this system has the advantage of being highly reliable and reproducible, unlike cell culture-based studies where CagA transfection is often toxic to cells and transfection rates are low. A complementary transgenic system for exploring CagA function has been developed in mouse [18]. This system demonstrated that CagA expression is sufficient to promote the development of gastrointestinal neoplasms with a low penetrance, however the host genetic pathways required for this process were not defined.

In this study, we uncovered a role for CagA in activating MLC. Using the Gal4-UAS system, we expressed CagA in the developing larval eye epithelium, a well-characterized model for epithelium formation, and found that CagA induced rapid epithelial disruption. We showed that reducing the levels of active MLC decreased the severity of epithelial disruption induced by CagA. In addition, we demonstrated that CagA causes increased phosphorylation and mispatterning of MLC. From these results, we conclude that MLC activation is a key target of CagA-induced epithelial disruption. Furthermore, we showed that the EPIYA motifs of CagA are necessary for proper apical targeting in the polarized retinal epithelial cells, and loss of these motifs renders CagA less potent in inducing morphological disruption in the epithelium. We showed that wild-type CagA and a CagA mutant lacking the EPIYA motifs are equally potent in inducing MLC redistribution in a cell type in which their subcellular localization patterns are equivalent, leading us to conclude that targeting of CagA to the apical domain is critical for efficient activation of MLC and subsequent epithelial disruption in the larval eye epithelium.

## Results

### CagA expression induces rapid epithelial disruption

Previous work in our lab showed that expression of CagA using the eye specific Gal4 line, GMR-Gal4, caused an adult “rough” eye phenotype [17], suggesting that CagA interferes with the processes required for the integrity of the eye epithelium. Multiple events, including cell fate misspecification, apoptosis, and improper early morphogenesis, can cause an adult “rough” eye [19]. Therefore, we sought to understand the developmental underpinnings of the CagA-induced adult “rough” eye phenotype.

**Figure 1:**
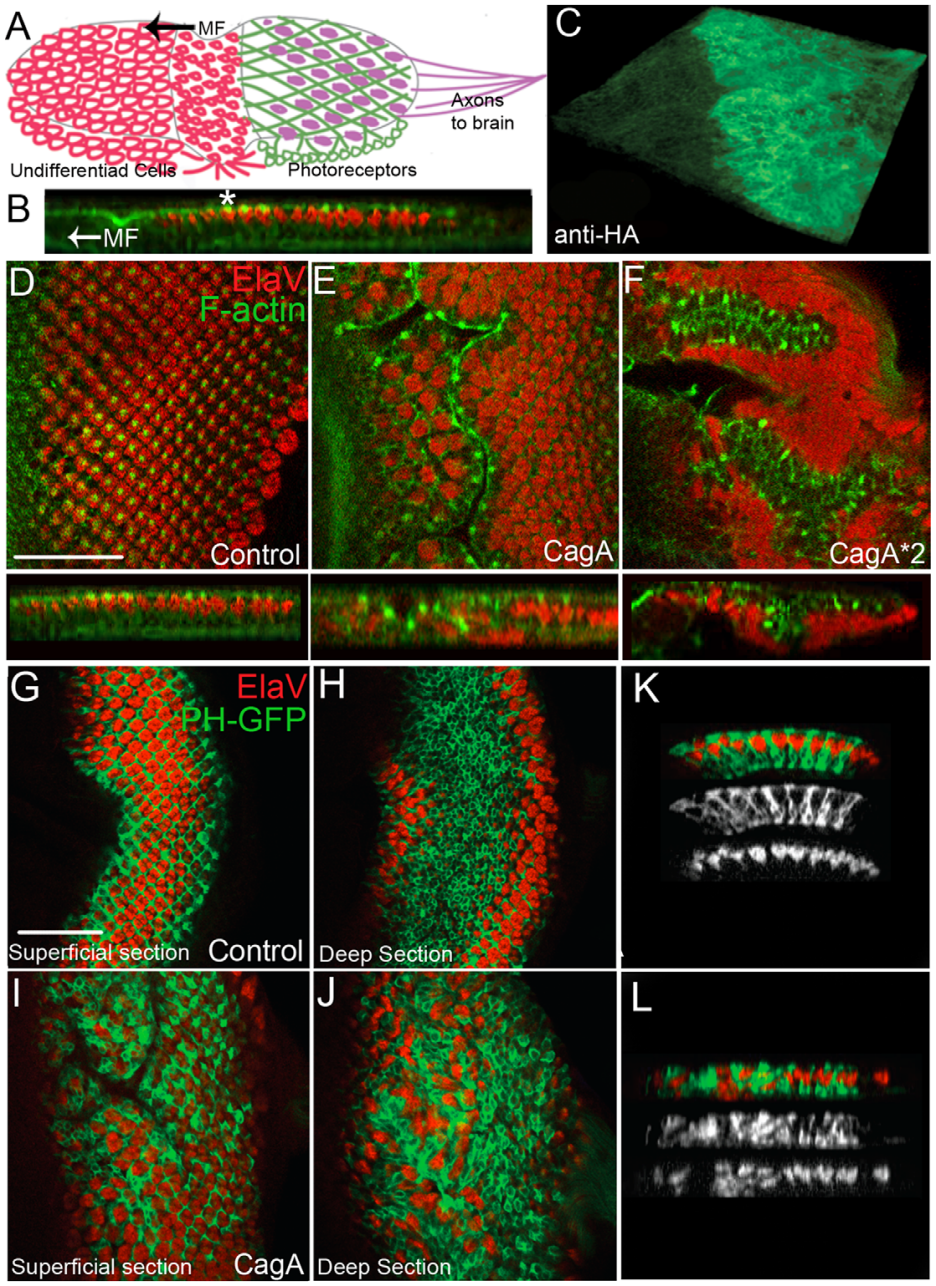
CagA induces rapid epithelial disruption. (A) Schematic of third instar larval eye disc development, showing the anterior advance of the morphogenetic furrow (MF). As the MF advances, undifferentiated cells (red cells on left) differentiate into photoreceptors (depicted as purple dots). (B) Cross section of a third instar larval epithelium labeled with F-actin (green) and ElaV (red) to mark photoreceptor nuclei. All images are oriented with anterior to the left. The MF advances in the direction of the arrow as development proceeds. The asterisk marks a punctum of actin apical to the photoreceptors. (C) A 3D reconstruction of a third instar larval eye disc expressing CagA-HA, as labeled by anti-HA. (D) XY confocal plane of a control eye epithelium expressing GMR-Gal4 alone. Photoreceptors (red) are spatially separated. The MF is positioned at the far left in D-F. Image below shows an optical cross section of a GMR-Gal4 eye epithelium showing planar arrangement of photoreceptor clusters (red) that each contact an apical actin punctum (green). (E) GMR-Gal4; UAS-CagA expressing eye epithelium displaying improper separation of actin foci into what appear at this resolution to be long bands of continuous actin. Lower panel shows a cross section of a GMR-Gal4;UAS-CagA expressing eye epithelium showing an ectopic furrow displacing photoreceptor nuclei basally. (F) GMR-Gal4; UAS-CagA*2 expressing eye disc displaying a deep ectopic furrow. Lower panel shows a cross section of a GMR-Gal4; UAS-CagA*2 expressing eye epithelium with photoreceptor nuclei displaced basally. (G) A superficial confocal plane of a GMR-Gal4 control eye disc showing PH-GFP expression surrounding ommatidia. (H) A deep confocal section showing the underlying photoreceptor cells and the absence of ElaV positive cells within the deep layers of the tissue. (I) Superficial confocal section of a GMR-Gal4; UAS-CagA eye epithelium (J) A deep section of a GMR-Gal4; UAS-CagA eye epithelium showing ElaV –positive cells within the deep region of the epithelium and interspersed with PH-GFP expressing cells. (K) An optical cross section through a GMR-Gal4 control eye epithelium showing the arrangement of PH-GFP and ElaV expressing cells. (L) An optical cross section through a GMR-Gal4; UAS-CagA expressing eye epithelium showing the apicobasal mispositioning of ElaV and PH-GFP expressing cells. Scale bars in D and G are 50 microns.

Because GMR-Gal4 drives expression initially in third instar larvae, we assessed morphological disruption at this stage. The eye imaginal disc is a pseudostratified epithelial monolayer comprised of undifferentiated cells that become photoreceptors whose nuclei are positioned apically, and support cells whose nuclei are positioned basal to the photoreceptors (Fig. 1A). An invagination in the disc, referred to as the morphogenetic furrow (MF), forms at the posterior end of the disc early in development and progresses anteriorly as development proceeds. Notably, MF formation requires proper regulation of myosin activity [14]. During the anterior progression of the MF, differentiation of photoreceptor cells, as marked by the neuronal marker ElaV (Fig. 1B), occurs posterior to the furrow. Differentiated photoreceptors form clusters, referred to as ommatidia. On the apical surface of each ommatidium is an actin-rich punctum, which co-stains with adherens junction markers. We confirmed that CagA was being expressed with the GMR-Gal4 driver, which drives expression in all cells posterior to the MF. Using an HA-antibody, we detected expression of CagA-HA throughout the differentiated eye epithelium (Fig. 1C).

To assess epithelial disruption, we examined F-actin expression and the position of photoreceptor nuclei in control and CagA-expressing eye epithelia. Control larval eye epithelia expressing only GMR-Gal4 display spatially separated ommatidia with actin foci at the apical cortex of each ommatidium (Fig. 1D). In controls, ommatidial nuclei are arranged within an apical plane (Fig. 1D). This planar arrangement is disrupted following CagA expression such that the position of photoreceptors is often shifted basally (Fig. 1E). This basal displacement can occur because the entire epithelium folds in on itself, forcing the photoreceptor nuclei beneath the normal plane of photoreceptors. We refer to this infolding process as ectopic furrowing. Basal nuclear displacement is also observed outside of ectopic furrows, which may reflect a failure of the photoreceptor nuclei to move apically, a process that normally occurs in photoreceptors immediately posterior to the furrow.

In contrast to the normal organization of discrete actin foci at the apical ommatidial cortex in control epithelia (Fig. 1B,1D), the spatial separation of actin foci was disrupted in CagA-expressing eye epithelia (Fig. 1E). This was particularly true within the ectopic furrows, where the actin foci on the apical membranes of several ommatidia appear to merge into a long band of actin expression (Fig. 1E). Eye discs expressing two copies of CagA displayed deeper and more extensive ectopic furrowing (Fig. 1F). In addition, photoreceptor nuclei lay predominantly along the basal surface of the epithelium (Fig. 1F). Therefore, the CagA-induced phenotype is dose-dependent.

To further characterize the apicobasal mispositioning elicited by CagA expression, we analyzed expression of the pleckstrin homology domain of Phospholipase C tagged with GFP (PH-GFP) driven with the GMR-Gal4 driver, which we fortuitously discovered to be an excellent marker for the deep support cells. In the apical regions of control eye epithelia, ElaV-expressing cells were observed in a patterned array (Fig. 1G). PH-GFP was expressed in a hexagonal pattern surrounding the ElaV-positive cells. In deep regions of the eye epithelium, ElaV cells were largely absent and PH-GFP cells predominated (Fig. 1H). In contrast, ElaV and PH-GFP cell did not display a similar pattern of apicobasal separation in CagA-expressing epithelia (Fig. 1I, 1J). In particular, ElaV-positive cells were frequently observed in deep regions of the eye epithelium and were intermingled with PH-GFP positive cells. This phenomenon was also apparent in orthogonal views of the eye epithelia. In controls, ElaV positive nuclei were present apically and PH-GFP nuclei were basally positioned (Fig. 1K). In CagA-expressing eye epithelia, ElaV positive nuclei were frequently observed at basal locations (Fig. 1L), highlighting the fact that CagA disrupts the normal organization of ElaV nuclei and the underlying support cells.

### CagA expression leads to misregulation of MLC

**Figure 2:**
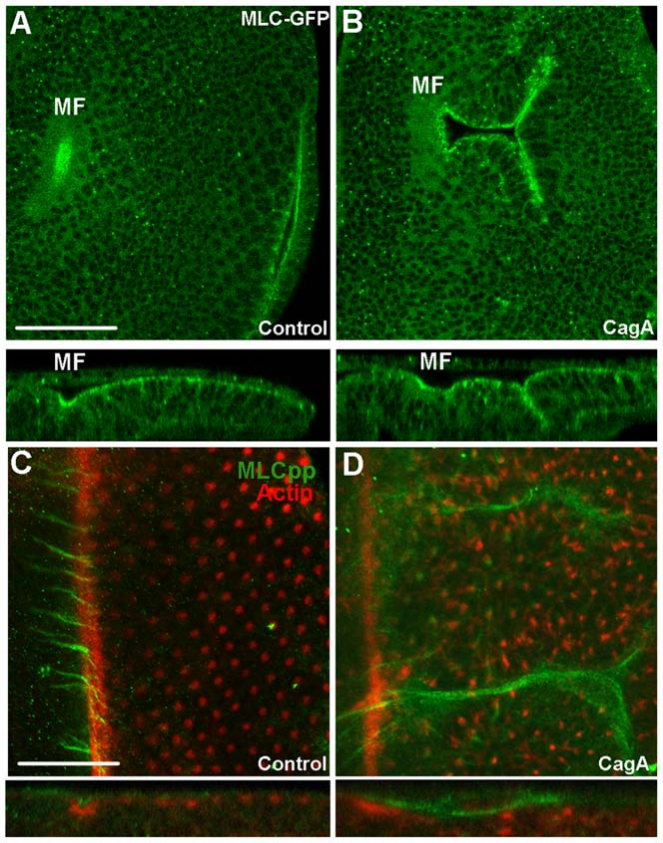
MLC regulation in CagA-expressing eye epithelia. (A) Deep confocal section of a control eye epithelium (GMR-Gal4) expressing MLC-GFP. Cross section reveals enrichment of MLC-GFP along the apical surface of the epithelium beginning at the MF. Scale bar is 50 uM. (B) Deep confocal section of a GMR-Gal4; UAS-CagA expressing eye epithelium. Confocal plane goes through an ectopic furrow revealing the infolded apical surfaces of the epithelium expressing enriched MLC-GFP. Cross section reveals apical MLC-GFP expression and enrichment of MLC-GFP in the ectopic furrow. (C) Flattened confocal stack of a control (GMR-Gal4) eye epithelium stained with anti-MLCpp (green) and phalloidin (red) to mark the MF. Cross section reveals MLCpp expression within the MF. Only very weak expression of MLCpp is present posterior to the furrow. Scale bar is 100 uM. (D) Flattened confocal stack of a CagA (GMR-Gal4; UAS-CagA) expressing eye epithelium displaying a complex pattern of MLCpp expression posterior to the MF. Optical cross section reveals MLCpp expression within an ectopic furrow.

Patterning of the complex epithelial architecture of the *D. melanogaster* eye epithelium is highly dependent on proper MLC regulation [14]. For example, formation of the MF requires MLC-dependent apical constriction [14]. Because of this central patterning role of MLC and because CagA caused ectopic furrows resembling the MF, we asked whether CagA expression alters the localization and activation of MLC. First, we assessed CagA’s ability to alter MLC-GFP expression to determine whether CagA influences MLC distribution. As previously observed, MLC-GFP expression was enriched in the apical domain of control larval eye epithelia [Fig. 2A; [14]]. In the CagA expressing epithelia, MLC-GFP was also apically distributed and appeared enriched in the ectopic furrows, although this could be due to the constriction of the apical surfaces of the cells within the invaginations (Fig. 2B).

In order to observe the active form of MLC, we took advantage of an antibody specific to *D. melanogaster* MLC when it is phosphorylated at both threonine-20 and serine-21. We refer to this form of MLC as MLCpp. In control eye epithelium, MLCpp is expressed strongly in arrays perpendicular and anterior to the MF, but weakly, in small foci posterior to it, suggesting that MLCpp is rapidly depleted in cells immediately posterior to the MF (Fig. 2C). In striking contrast to control eye epithelia, MLCpp expression in CagA expressing eye epithelia is highly enriched in a pattern that extends posterior to the MF (Fig. 2D). Thus, we conclude that CagA either blocks depletion of MLCpp posterior to the MF or ectopically activates MLC posterior to the MF.

### CagA-induced epithelial disruption is suppressed by reducing active MLC

**Figure 3:**
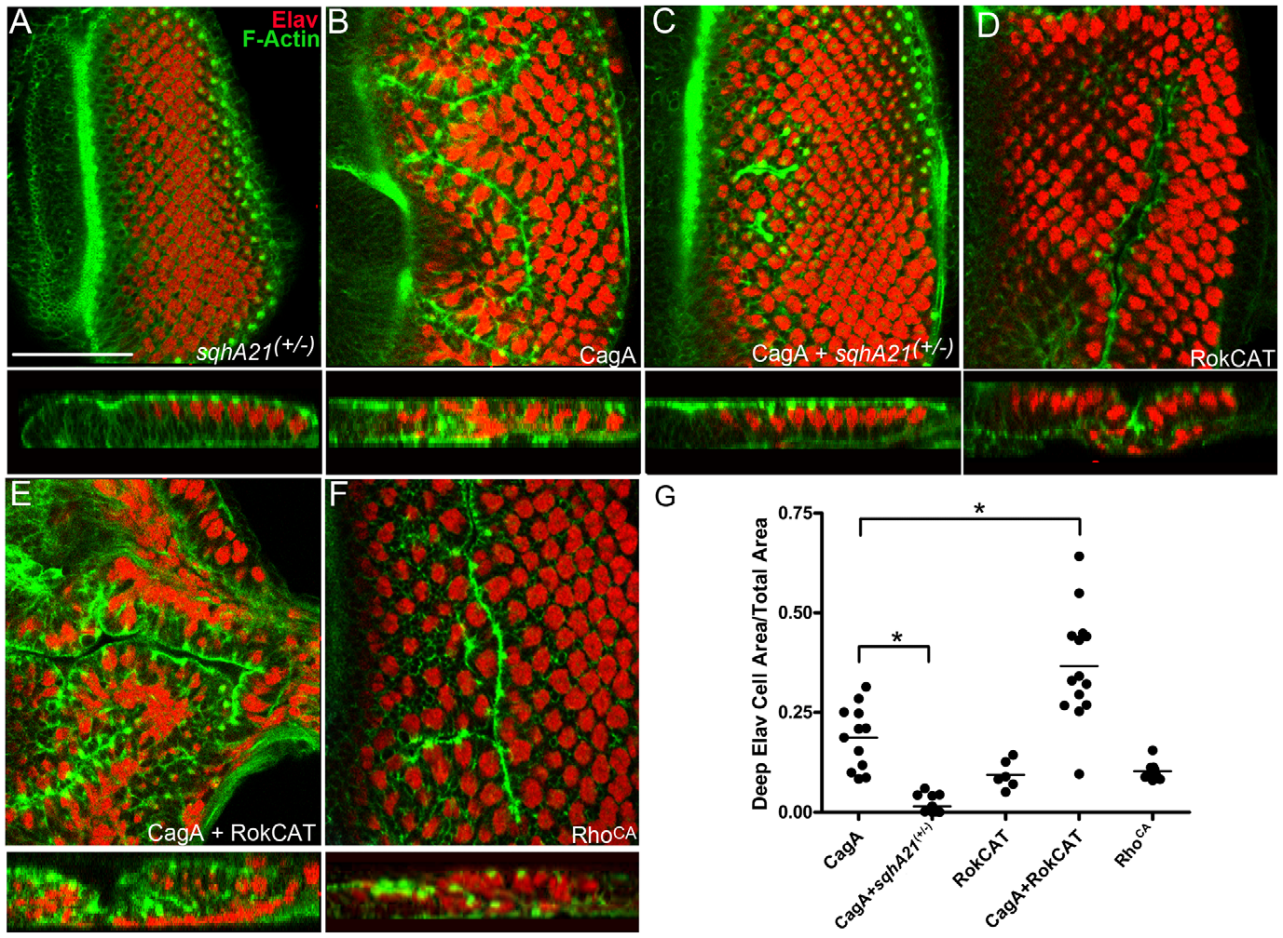
CagA interactions with MLC and the MLC activator, Rok. (A) Eye epithelium expressing an inactivating MLC mutation (*sqhA21*) displaying normal morphology. ElaV (red) labels photoreceptors and phalloidin (green) labels F-actin. Scale bar is 50 microns. (B) GMR-Gal4; UAS-CagA expressing eye epithelium displaying ectopic furrowing. (C) GMR-Gal4;UAS-CagA expressing eye epithelium co-expressing a single copy of *sqhA21* displaying very mild ectopic furrowing. (D) GMR-Gal4; UAS-RokCAT epithelium with ectopic furrows. (E) GMR-Gal4; UAS-CagA and UAS-RokCAT displaying severe ectopic furrowing. (F) GMR-Gal4; UAS-Rho^CA^ displaying moderate ectopic furrowing. (G) Quantification: The area of deep photoreceptors was determined by first inverting a 3D reconstruction of each eye epithelium, and determining the area of ElaV expression in Image J. This value was divided by the total area of the eye epithelium, thus providing a metric for morphological disruption. * indicates statistical significance. P value for CagA vs CagA; sqhA21^(-/-)^ is less than 0.0001, and for CagA vs CagA; RokCAT p value is 0.0005.

Because we observed that CagA influences the pattern of activated MLC, we asked whether modulating the amount of available active MLC would alter the disruption induced by ectopic CagA expression in the eye epithelium. We used a dominant negative *mlc* transgenic allele (*spaghetti squash A21*; *sqhA21*)) in which one of the two phosphorylation sites on MLC is mutated, rendering it unphosphorylateable at this site [15]. We asked whether the expression of this inactivatable form of MLC would lessen the disruption caused by CagA expression. To assess the degree of morphological disruption, we examined the pattern of actin and ElaV expression in each eye epithelium. In *sqhA21* mutants, no discernible actin mispatterning defect or ElaV mispositioning was observed (Table 1, Fig. 3A), consistent with a previous report [20]. As we observed in Fig. 1E, ElaV positive cells and actin foci were basally displaced in CagA-expressing eye epithelia epithelium (Fig. 3B). However, morphological disruption was greatly reduced when CagA was co-expressed with a single copy of the inactivating *sqhA21* mutation (Fig. 3C). In these epithelia, ElaV-positive cells and actin foci were predominantly apically positioned (Fig. 3C), which more closely resembled the morphology of control eye epithelia (Fig. 1D). To quantify the degree of rescue, we divided the total area of deep ElaV positive expression by the total area of the differentiated eye epithelium. Using this metric, we demonstrated a statistically significant rescue of CagA morphological disruption by co-expression of *sqhA21* (Fig. 3G). In order to elevate MLC activity, we expressed the catalytic domain of Rho kinase (RokCAT), which phosphorylates and activates MLC, using GMR-Gal4 [13], [21]. On its own, RokCAT expression caused mild ectopic furrowing (Fig. 3D, 3G; Table 1). In contrast, epithelia co-expressing both CagA and RokCAT had a significant increase in basally positioned ElaV-positive cells and actin foci (Fig. 3E, 3G). Our results demonstrated that CagA-induced epithelial disruption is reduced or enhanced by modulating the amount of active MLC.

**Table 1:** Characterization of genetic manipulations of the cytoskeleton in the eye disc.

Genotype	Eye expression phenotype	Known Cellular Function
GMR-Gal4;UAS-Rho^CA^	Basal displacement of Elav cells; ectopic invaginations	Small RhoGTPase; cytoskeletal regulator
GMR-Gal4; UAS-Rho^DN^	Basal displacement of Elav cells; ectopic invaginations	Small RhoGTPase; cytoskeletal regulator
GMR-Gal4; UAS-RokCAT	Basal displacement of Elav cells; ectopic invaginations	Rho-assocaited protein kinase. Seri/Thr kinase; activator of MLC
GMR-Gal4; UAS-RhoGEF2	Mild ectopic invaginations, some basally displaced ElaV cells.	Guanine nucleotide exchange factor for Rho1.
GMR-Gal4; UAS-MLCK-CT	Broad epithelial disruption	myosin activating kinase
GMR-Gal4; UAS-Cdc42^CA^	Broad epithelial disruption; loss of adherens junction	Small RhoGTPase; cytoskeletal regulator
GMR-Gal4: UAS-Rac^CA^	Broad epithelial disruption; loss of adherens junction	Small RhoGTPase; cytoskeletal regulator
GMR-Gal4; UAS-Rac^DN^	No phenotype	Small RhoGTPase; cytoskeletal regulator
GMR-Gal4; UAS-Dia^CA^	Basal displacement of ElaV cells; loss of adherens junctions	Formin; Rho effector
gmrGal4; UAS-Csw^src90^	No phenotype	Constitutively active SHP2 phosphatase; oncoprotein; known CagA interactor
gmrGal4; UAS-Csw^D545A^	No phenotype	Dominant negative SHP2
*par1* ^ (+/−)^	No phenotype	Polarity mediator; kinase shown to interact with CagA
GMR-Gal4; UAS-CagA + *par1* ^(+/−)^	Strong enhancement of CagA phenotype	

### Activation of Rho pathway members causes similar disruption as CagA

We surveyed a collection of cytoskeletal interactors to determine whether the epithelial disruption induced by CagA is a specific response to MLC regulators or whether it can be caused by more generally disrupting the epithelial cytoskeleton. We found that, when expressed in eye epithelia, a RhoA constitutively active mutant (RhoA^V14^), which we refer to as Rho^CA^, displayed ectopic furrows highly similar to those induced by CagA expression (Fig. 3F). We observed similar disruption by expressing RhoGEF2 (Table 1), a guanine nucleotide exchange factor that activates Rho. As mentioned earlier, RokCAT also caused ectopic furrowing (Fig. 3D; Table 1). Surprisingly, a dominant negative form of RhoA, Rho^N19^, also caused a similar disruption of the eye epithelium as CagA (Table 1). We speculate that this is due to improper cycling of the RhoGTPase. In contrast, other Rho GTPases known to regulate the cytoskeleton, such as Cdc42 and Rac, as well as factors known to regulate actin dynamics, such as slingshot and diaphanous, caused distinct phenotypes (Table 1). Constitutively active MLCK caused severe disruption of the eye epithelia, which made comparison with CagA difficult (Table 1). Although loss of a single copy of the polarity mediator and known CagA interactor, *par 1*, did not cause a discernible eye epithelium phenotype by itself, *par1* ^(+/-)^ flies expressing CagA displayed enhanced disruption when compared to eye epithelia expressing only CagA (Table 1). Interestingly, a recent report using polarized culture cells showed that expression of Par1b blocks MLC activity in Rho-dependent fashion [22]. Therefore, loss of *par1* may lead to heightened MLC activity in the eye epithelium, thus enhancing CagA’s ability to disrupt the epithelia.

### CagA activates MLC in a tissue culture model

**Figure 4:**
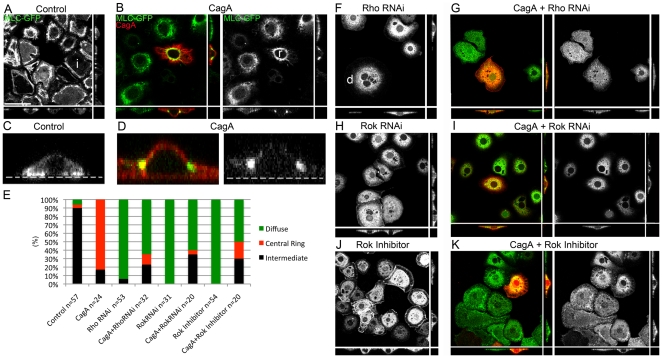
CagA induces Rho-dependent MLC-GFP relocalization in S2 cells. (A) Control S2 cells plated on ConA expressing MLC-GFP predominantly in the periphery of cell. Cell labeled i is representative of the intermediate phenotype. Scale bar: 25 microns. (B) A CagA transfected S2 cell expressing MLC-GFP in a central ring surrounding the nucleus. Cell labeled r is representative of the central ring phenotype. (C) High magnifcation optical cross section of a control MLC-GFP expressing S2 cell showing MLC-GFP expression in close association with the substrate (labeled with a transparent line). (D) High magnification optical cross section of a control MLC-GFP expressing S2 cell showing MLC-GFP expression above the substrate. (E) Graph showing the relative distribution of cell types (diffuse, central ring, or intermediate) in each treatment. (F) A Rho RNAi treated S2 cell showing diffuse MLC-GFP expression. Cell below d is representative of the diffuse phenotype. (G) A CagA transfected cell treated with Rho RNAi showing diffuse MLC-GFP expression. (H) A Rok RNAi treated S2 cell showing diffuse MLC-GFP expression (I) A CagA transfected cell treated with Rok RNAi showing diffuse MLC-GFP expression. (J) A Rok inhibitior (Y-27632)-treated cell showing diffuse MLC-GFP expression. (K) A Rok inhibitor treated cell showing diffuse MLC-GFP expression.

Our work in the eye epithelium argued that CagA induces morphological disruption by activating the Rho/MLC pathway. To further characterize the functional interaction between CagA, Rho and MLC, we asked if CagA expression altered the subcellular localization of MLC in S2 cells, which are stable cells derived from *D. melanogaster* hemocytes. When plated on Concanavalin A (ConA), S2 cells acquire a flattened morphology that is amenable to high-resolution imaging (Fig. 4A). Upon expression of Rho^CA^ and RhoGEF2, a radical redistribution of MLC-GFP occurs in S2 cells cultured on ConA [23]. Specifically, activation of the Rho pathway causes the majority of MLC-GFP signal to localize to a central ring, where it associates with RhoGEF2 and Rho [23]. Because of this association with RhoGEF2, it is inferred that the majority of MLC within the central ring is phosphorylated and hence active [23].

We were able to transfect S2 cells with CagA, albeit at an inefficient rate (approximately 1 in 200 cells), and found that CagA induced similar redistribution of MLC-GFP as seen in cells expressing activated Rho or RhoGEF2. The induction of a central ring of MLC-GFP expression in the S2 cell model provided us with a straightforward read-out of CagA’s ability to activate MLC. To compare MLC-GFP expression across different treatments, we classified cells into 3 phenotypic categories. Cells displaying MLC-GFP throughout the cell were labeled as “diffuse.” “Intermediate” cells displayed MLC-GFP expression predominantly around the periphery of the cell and in intimate association with the underlying substrate. “Central ring” cells displayed MLC-GFP expression in a tight ring around the nucleus or immediately adjacent to it. Control cells predominantly displayed the intermediate phenotype, however, a minority of cells displayed largely diffuse cytoplasmic staining (Fig. 4A, E). In CagA expressing cells, we observed a tight ring of expression either surrounding the nucleus (as in Fig. 4C), or adjacent to it. Similar to RhoGEF2 or activated Rho expressing S2 cells, MLC-GFP was positioned above the membrane contacting the underlying substrate in CagA expressing cells (Fig. 4D).

To determine if CagA indeed requireed Rho and Rok to redistribute MLC, we used RNA interference (RNAi) to knock down expression of Rho and Rok, as well as a chemical inhibitor of Rok, Y-27632, to determine if CagA was able to redistribute MLC-GFP even in cells depleted of Rho and Rok activity. Rho RNAi on its own caused MLC to become highly diffuse throughout the cell [Fig. 4E,F [23]]. When CagA was expressed in Rho RNAi treated cells, MLC-GFP remained diffuse in the majority of instances (Fig. 4G), demonstrating that CagA was not able to redistribute MLC-GFP in the absence of Rho. Likewise, Rok RNAi treatment caused MLC-GFP to become diffuse throughout the cell (Fig. 4E,H). CagA was significantly less effective in redistributing MLC-GFP in cells treated with Rok RNAi (Fig. 4E, I). Similarly, blockade of Rok activity with the chemical inhibitor, Y-23762 (Fig. 4E, J), caused MLC-GFP to become highly diffuse, and CagA was dramatically impaired in its ability to redistribute MLC-GFP in Y-23762-treated cells (Fig. 4E, K). From these results we concluded that CagA activates MLC via a mechanism that requires Rho and Rok.

### CagA^EPISA^ is a less potent epithelial disruptor than CagA

**Figure 5:**
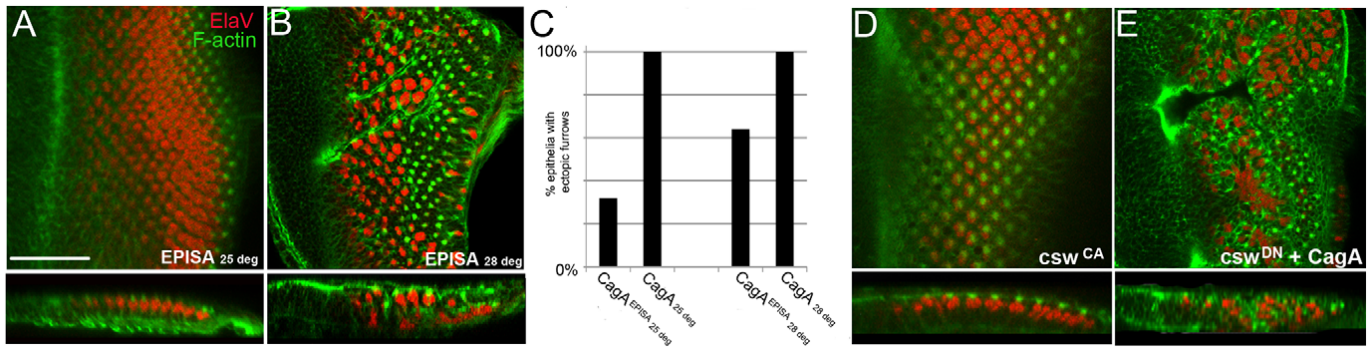
Efficient epithelial disruption requires EPIYA motifs but not SHP2/Csw interaction. (A) GMR-Gal4; UAS-CagA^EPISA^ raised at 25 degrees C displaying no overt signs of disrupted morphogenesis. Elav (red), F-actin (green) (B) GMR-Gal4; UAS-CagA^EPISA^ raised at 28 degrees C displaying ectopic furrowing. (C) Comparison of frequency of ectopic furrowing between wild-type CagA and CagA^EPISA^ at different temperatures. (D) GMR-Gal4; UAS-csw^src90^ displaying no signs of morphological disruption. (E) GMR-Gal4; UAS-csw^DN^ does not rescue CagA-induced furrowing.

To investigate the mechanism by which CagA activates MLC, we asked whether a mutant form of CagA that lacks sites for tyrosine phosphorylation (termed CagA^EPISA^) is capable of inducing morphological disruption. CagA phosphorylation occurs within EPIYA motifs, and these repeated motifs have been shown to mediate diverse biological processes, particularly SHP-2-mediated cell elongation [5]. We asked whether CagA^EPISA^ is able to cause disruption of the larval eye epithelium. Previously, we showed that CagA^EPISA^ does not cause “roughness” in adult eyes (Botham, 2008). In larvae raised at 25 degrees C, we observed that CagA^EPISA^ caused ectopic furrowing, but did so only in 32% of cases (Fig. 5A). Taking advantage of the temperature dependency of the Gal4-UAS system, we asked whether raising temperature would enhance CagA^EPISA^’s ability to induce morphological disruption. Enhanced transcription occurs at higher temperature because of enhanced stability of the Gal4 transcription factor. We found that in 64% of cases, CagA^EPISA^ was able to induce ectopic furrowing at 28 deg (Fig. 5B). However, CagA^EPISA^ was significantly less potent than wild-type CagA in causing ectopic furrowing. In fact, wild-type CagA induced ectopic furrowing in 100% of the eye epithelia studied, even at 25 degrees C (Fig. 5C). Therefore, the EPIYA motifs enhance CagA’s ability to disrupt the epithelium. However, the EPIYA motifs are not necessary for epithelial disruption because when they are not present, as in CagA^EPISA^, epithelial disruption still can occur.

### SHP-2, a well-characterized target of CagA, does not enhance CagA-induced epithelial disruption

Activation of SHP-2 by CagA requires functional EPIYA motifs and SHP-2 activation has been shown to be critical in the cytoskeletal disruption that occurs during *H. pylori* infection of cultured human gastric cells [24]. Our results demonstrate that efficient epithelial disruption by CagA depends on the presence of the EPIYA domains. Therefore, we asked whether the impaired ability of CagA^EPISA^ to disrupt epithelia was due to CagA^EPISA^’s inability to activate SHP-2. We found that expressing an activated form of the *D. melanogaster* SHP-2 homolog, *corkscrew*, (*csw*), which is targeted to the membrane via an engineered myristoylation site [25], did not induce the cytoskeletal abnormalities observed in CagA-expressing eye epithelium (Fig. 5D). In addition, we co-expressed CagA and an engineered dominant negative form of Csw (Csw^DN^) containing an inactivating mutation in the phosphatase domain that is predicted to create a dominant negative form of the protein (Csw^DN^)[26]. On its own, Csw^DN^ did not cause morphological disruption at larval stages, but did cause a mild rough eye phenotype in adults (not shown). When co-expressed with CagA, we found that this phosphatase-dead Csw mutant did not reduce the degree of epithelial disruption caused by CagA expression (Fig. 5E). Therefore, Csw activation is not sufficient or necessary for CagA-induced epithelial disruption in the larval eye epithelium.

### CagA EPIYA motifs are necessary for apical localization of CagA

**Figure 6:**
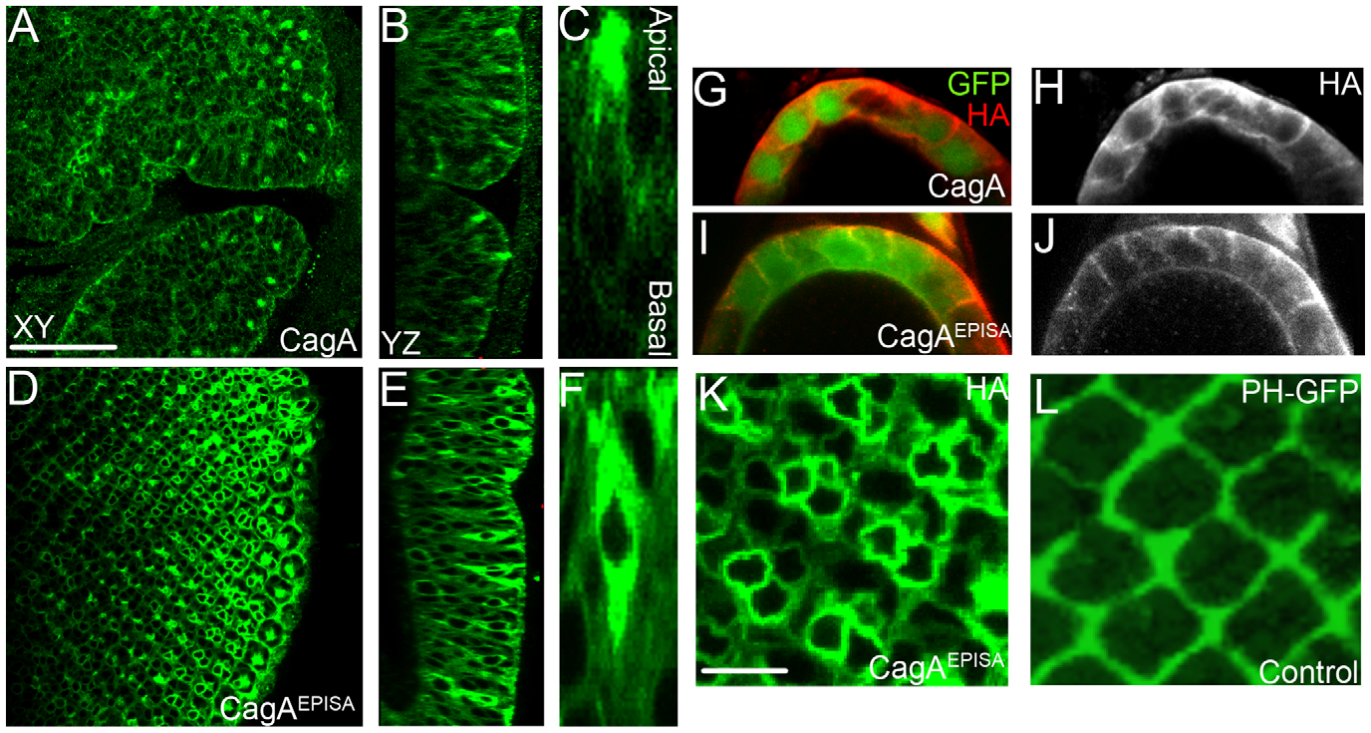
CagA localization is directed by EPIYA motifs. (A) GMR-Gal4; UAS-CagA expressing eye epithelium labeled with anti-HA to reveal the pattern of CagA expression. Scale bar for A, B, D, E: 50 microns. (B) YZ optical section of (A) showing HA expression in apical punctae. (C) A high magnification view of (A) showing an ommatidium with HA expression concentrated at the apical foci. (D) GMR-Gal4; UAS-CagA^EPISA^ expressing eye epithelium. (E) YZ optical section of (D) showing diffuse, cytoplasmic expression in individual ommatidial cells. (F) A high magnification view of (E) showing HA expression throughout the cell. (G) *slbo*-Gal4; UAS-GFP, UAS-CagA expressing follicular epithelial cell. (H) Cortical enrichment of HA expression in follicular epithelial cells. (I) *slbo*-Gal4: UAS-GFP, UAS-CagA^EPISA^ expressing follicular epithelial cells. (J) Cortical enrichment of HA expression in CagA^EPISA^ expressing follicular epithelial cells. (K) GMR-Gal4; UAS-CagA^EPISA^ expressing eye epithelium showing high HA expression in a subset of photoreceptors. (L) GMR-Gal4; UAS-PH-GFP expressing eye epithelium showing GFP expression surrounding the ommatidia but not in photoreceptors. Scale bar in K and L: 10 microns.

EPIYA domains have been shown to target CagA to the membrane of human gastric cells [27]. Therefore, we asked whether the EPIYA motifs influence CagA subcellular localization in our model. We found that CagA^EPISA^ was robustly expressed in morphologically normal epithelia at levels roughly equivalent to wild-type CagA (Fig. 6A,D), consistent with our previous report that CagA and CagA^EPISA^ are expressed at similar levels, as evaluated by Western blots [17]. However, we noticed a striking difference in the subcellular localization of CagA and CagA^EPISA^. We found that CagA expression was enriched in apical foci of cells we interpret to be photoreceptors due to their apical position within the epithelium (Fig. 6B,C). This was in contrast to CagA^EPISA^ which was uniformly expressed throughout the cytoplasm of a subset of photoreceptors (Fig. 6E, F). Therefore, the EPIYA domains target CagA to the apical domain of the photoreceptors.

To determine if the EPIYA motifs localize CagA in other tissues, we expressed CagA in the posterior follicular epithelium of the fly ovary. We observed that CagA expression was cortically enriched in these cells (Fig. 6G, H). In these cells, CagA^EPISA^ was also cortically enriched (Fig. 6I, J). Therefore, the EPIYA motifs are not necessary for cortical localization in all epithelia.

In addition to EPIYA motifs, a phosphatidyl serine (PS) binding motif within CagA has been shown to direct CagA to the membrane [28]. In polarized MDCK cells, interaction between CagA and PS is sufficient to tether CagA to the membrane even in the absence of EPIYA motifs [28]. The PS binding motif of CagA has been mapped to a consensus sequence found in pleckstrin homology (PH) domains that is responsible for specific phospholipid binding. The PH domain of Phospholipase C gamma has a similar PS-binding consensus motif as CagA. By expressing a GFP-tagged form of PH (PH-GFP), we predicted that if it is indeed the case that EPIYA and PS-binding motifs are the primary determinants of CagA’s subcellular localization, then this fusion protein should mimic CagA^EPISA^ expression. Like CagA^EPISA^, PH-GFP expression was observed in the hexagonal array of cells surrounding the ommatidia, and was absent from the apical foci of photoreceptor cells (Fig. 6K, L). However, in contrast to PH-GFP, CagA^EPISA^ was also strongly expressed cytoplasmically in a pair of cells within the ommatidium (Fig. 6L). Therefore, PH-GFP expression was not an absolute predictor of CagA^EPISA^ expression in the larval eye epithelium.

### CagA and CagA^EPISA^ are equally potent activators of MLC in cultured cells

**Figure 7:**
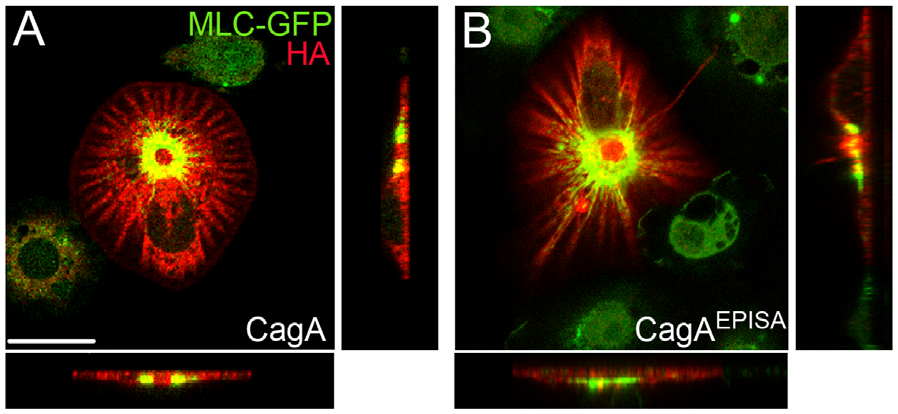
CagA and CagA^EPISA^ expression in MLC-GFP-expressing S2 cells. (A) CagA expressing S2 cell marked with HA antibody (red). In the transfected cell, MLC-GFP (green) is expressed in a “central ring.” (B) A CagA^EPISA^ expressing cell showing the central ring MLC-GFP pattern. Scale bar: 5 microns.

The complexity of the larval eye epithelium made it difficult to predict CagA localization. We asked whether CagA and CagA^EPISA^ had similar subcellular localization in a simpler context, S2 cells. In examining the expression patterns of CagA^EPISA^ and CagA, we found that both are expressed throughout the cortex and cytoplasm (Fig. 7A, B). CagA^EPISA^ and CagA expressing cells have a stellate morphology (Fig. 7A, B), a pattern that likely results from microtubule polymerization stimulated by the Rho activator, RhoGEF2 [23]. Therefore, in the context of S2 cells, CagA and CagA^EPISA^ have equivalent expression patterns, unlike in the larval eye epithelium. We asked whether CagA was more potent than CagA^EPISA^ in eliciting the central ring of MLC-GFP in S2 cells. Upon expression of CagA^EPISA^ in S2 cells, MLC-GFP was distributed in a central ring in the majority of transfected cells (Fig. 7B). In fact, we found that CagA^EPISA^ was equally as potent as CagA in inducing the MLC-GFP central ring pattern. 85% (n = 14) of CagA^EPISA^ transfected S2 cells displayed the central ring phenotype versus 87% (n = 24) of CagA transfected S2 cells. Therefore, we argue that subcellular localization of CagA, which varies as a function of CagA sequence and cellular context, is a critical factor in determining the potency of CagA’s activation of MLC.

## Discussion

In this study, we used a transgenic *D. melanogaster* model to elucidate a role for *H. pylori* CagA in activating MLC and inducing epithelial disruption. CagA caused a characteristic disruption of the eye epithelium that corresponded to elevated MLC phosphorylation levels and was suppressed by co-expression of an inactivating MLC mutation. In S2 cells, CagA activated MLC in a Rho and Rok dependent manner. We asked whether CagA’s ability to influence MLC activity is dependent on the phosphorylation of the EPIYA domains of CagA and found that CagA^EPISA^ has impaired capacity to disrupt epithelia. We showed that the reduced potency of the CagA^EPISA^ mutant could not be explained by a role for SHP-2/Csw in the morphological disruption. Instead, we argue that this impairment is due to CagA^EPISA^’s failure to concentrate in the apical domain of the larval eye epithelia, because in culture cells in which subcellular localization patterns of CagA^EPISA^ and CagA are the same, CagA^EPISA^ and CagA are equally potent in activating MLC.

Based on work in cultured cells, SHP-2 is thought to be a primary mediator of cytoskeletal disruption induced by CagA [24]. However, in a previous report, our lab showed that CagA-induced cell elongation in cultured human gastric cells results from a failure of cell retraction, a process typically modulated by Rho activity at the trailing edge [8], [9]. In the *D. melanogaster* model we used in this study, unlike in cultured cells, we were able to directly test functional interactions between CagA and the Rho/MLC pathway. In this intact epithelial tissue, we find that the Rho/MLC pathway is the critical mediator of cytoskeletal disruption, whereas SHP-2 activation caused no epithelial disruption in larval stages, and SHP-2 inactivation failed to rescue CagA-induced epithelial disruption. Therefore, by examining CagA function within an intact epithelium, our study reveals Rho/MLC as a critical host effector of CagA-induced epithelial disruption.

Recent studies have suggested that *H. pylori* activates MLC in host cells. However, the connection between CagA and MLC remains controversial. In one report, it was shown that blocking MLC activity with blebbistatin exacerbates CagA-induced cell elongation [29]. From this, the authors concluded that CagA downregulates MLC activity. On the other hand, two other recent studies that used cultured epithelial monolayers showed that *H. pylori* infection leads to activation of MLC [30], [31]. In one of these reports, interleukin 1 receptor signaling was found to be critical for MLC activation [31], and in the other, urease was implicated in MLC activation [30]. Both studies argued that CagA was not involved in activation. These results highlight the fact that bacterial infection leads to complex physiological responses in host cells, and deciphering the role of a single factor involved in infection is not always straightforward. Through our reductionist approach in which we study CagA function outside of the context of infection, we show that CagA by itself is sufficient to activate MLC. Supporting this conclusion are our observations that blocking myosin activity rescues the morphological defects elicited by CagA expression, and that in S2 cells CagA expression redistributes MLC in a manner similar to Rho activation. Therefore, CagA likely acts in concert with other factors involved in *H. pylori* infection, such as IL-1R and urease, to cause maximal MLC activation.

The results of this study demonstrate a correlation between CagA localization and potency of epithelial disruption. However, the mechanisms that target CagA intracellularly are not fully understood. We show that the EPIYA motifs are critical for targeting CagA to the apical foci of epithelial cells in the retina. Previously, it was shown that EPIYA motifs are critical for membrane localization in cultured human gastric cells [27]. However, in polarized canine kidney cells, a C-terminal construct containing EPIYA motifs was diffusely expressed in the cytoplasm, highlighting the fact that CagA localization is highly context specific [6]. In this same model, the N-terminal domain alone was targeted to the membrane, suggesting the presence of a membrane-targeting motif within the N-terminus [6]. Recently it was found that targeting of the N-terminus to the membrane in polarized MDCK cells requires the presence of a PS binding motif [28]. Therefore, in certain contexts, both EPIYA domains and the PS binding domain direct CagA to the membrane. However, CagA localization cannot always be predicted solely on the presence or absence of these domains. For example, in polarized cells, a CagA variant (CT 550-1216) that has both the EPIYA domains and the PS-binding domain is diffusely expressed throughout the cytoplasm [6]. Further highlighting the difficulty in predicting CagA localization, we predicted that PH-GFP would localize to the same region of the eye epithelium as CagA^EPISA^, because CagA^EPISA^ only has the PS binding domain. However, this is not what we observed. PH-GFP and CagA^EPISA^ have different patterns of localization suggesting that there are other factors that determine whether CagA is directed to the membrane besides the EPIYA motifs and the PS-binding motif. Other domains within CagA that have not yet been characterized may be critical for membrane localization. Additionally, interactions between CagA domains and host proteins specific to different cell types may be critical for determining localization. This point is reinforced by our observation that CagA localizes differently in the follicular epithelium of the ovary versus the larval eye epithelium. We speculate that the reason for this difference is that the mechanisms that position junctional proteins within the follicular epithelium are distinct from those acting in the larval eye epithelium [32]. During the development of the follicular epithelium, physical contact with germ cells is required for apical localization of the polarity determinant Crbs, which in turn positions the junctional component, Discs lost. In contrast, the apical positioning of junctional components in the larval eye epithelium is dependent, at least in part, on myosin activation [14]. Myosin activation is also critical for positioning of tight junction components in cell monolayers derived from the human intestine [33]. Therefore, we speculate that the larval eye epithelium more closely approximates how CagA behaves in the human stomach as compared with the follicular epithelium.

Although the *D. melanogaster* larval eye is a highly specialized epithelium and is in many ways unlike CagA’s normal milieu, the human stomach, we found the *D. melanogaster* larval eye to be highly responsive to CagA. What likely contributes to CagA’s effectiveness in disrupting our model epithelium is the dynamic nature of apical MLC within it. Networks of apical MLC are a feature of most epithelia, and upregulation of apical MLC has been shown to be clinically significant. For example, increased levels of apical MLC and MLCK in enterocytes of the human intestine correlate with the severity of Intestinal Bowel Disease (IBD) [34]. In addition, apical MLC concentrates at wound sites in human intestinal biopsies [35]. We observe that wild-type CagA is enriched apically in the larval eye epithelium due to targeting by the EPIYA motifs, and that this apical enrichment correlates with severity of epithelial disruption. Understanding the mechanisms by which EPIYA motifs target CagA to the apical surface of the epithelium will be of critical importance in determining the degree to which *H. pylori* influences MLC activity within the human gastric epithelium.

Both *H. pylori* infection and chronic MLC activation by transgenic MLCK expression lead to broad immune activation through enhanced paracellular flux and heightened pro-inflammatory cytokine expression [16], [36]. This alone does not necessarily cause disease; chronic MLC activation leads to subclinical outcomes, and the majority of *H. pylori* infected patients do not develop peptic ulcers or cancer [16]. It has been proposed, however, that each condition creates an environment in which the epithelium is more sensitive to additional cancer-promoting insults [1], [16]. In the case of *H. pylori*, these additional insults include activation of the oncogene SHP-2, impairment of the polarity protein, Par-1, and activation of the epidermal growth factor (EGF) signaling [5], [37].

## Materials and Methods

### Fly stocks and husbandry

The following fly lines were used: GMR-Gal4, UAS-CagA (Botham et al. 2008), UAS-RhoV14 (Bloomington Stock Center, Stock #8144), UAS-RhoN19 (Bloomington Stock #7327), UAS-csw^src90^ and UAS-csw^D545A^ (provided by Lizabeth Perkins): UAS-Cdc42.V12 (Bloomington Stock Center, Stock #6287), UAS-Rac1.N17 (Bloomington Stock #6292), UAS-Rac1.V12 (Bloomington Stock #6291), UAS-RokCAT (Bloomington Stock #6669), UAS-RhoGEF2 (Bloomington Stock #9386), UAS-MLCK-CT (provided by M. Van Berkum, Wayne State), UAS-Dia^CA^ (Bloomington Stock #27616), UAS-Ssh (Bloomington Stock #9114), sqhA21 (provided by Liquin Luo, Stanford). Stocks were provided by the Bloomington Stock Center unless otherwise noted above. Flies were raised at 25 degrees (unless otherwise noted) using standard techniques.

### Antibodies and Staining Procedures

Phalloidin and antibody staining of eye imaginal discs, egg chambers and S2 cells was carried out by standard techniques and with the following antibodies: *D. melanogaster* anti-Elav (1∶40; provided by C. Doe, University of Oregon), mouse anti-HA (1∶100; Covance), *D. melanogaster* ppMLC (1∶100; provided by Robert Ward, University of Kansas). Tissues were fixed in 4% Paraformaldehyde (30 minutes for eye discs and S2 cells (except for ppMLC staining which was 27 minutes), 20 minutes for egg chambers), blocked in PBSBT for 1 hr, and overnight primary antibodiy incubation at 4 degrees overnight was following by secondary antibody incubation overnight. Phalloidin 488 (1∶100; Life Technologies) was added to the secondary antibody incubation.

### Imaging and Image Analysis

Images were collected with a Nikon confocal microscope. The 3D reconstruction in Fig. 1C was made in Volocity (Improvision). To quantify the degree of morphological disruption in eye epithelia, confocal stacks were imported into Image J (NIH), 3D reconstructions were made using Volume Viewer, and images were thresholded to gain area measurements of deep ElaV cells. Unpaired t tests with Welches’ correction were used to determine statistical significance.

### Cell Culture, RNAi and Transfection

Drosophila S2 cell maintenance was performed as described previously. MLC-GFP cells were obtained from the Drosophila Genomics Resource Center. RNAi was performed according to published methods using previously reported sequences for template generation [23]. S2 cells were transfected with pMT-CagA or pMT-EPISA using Fugene (Roche) transfection reagent. CagA and CagA^EPISA^ (provided by H.Higashi) were cloned into pMT vector using standard cloning procedures. After 24 hours of incubation with the transfection complex, CagA expression was induced with copper sulfate (1 mM) for 24 hours. For experiments involving combined transfection and RNAi treatment, transfection was performed 48 hours after addition of RNAi and cells were fixed at 72 hours of RNAi treatment. Y-27632 was purchased from Calbiochem and used at 100 uM.
